# Quality and Accuracy of Information Available on Websites for Distracted Driving: Qualitative Analysis

**DOI:** 10.2196/16154

**Published:** 2019-12-20

**Authors:** Jeffrey Poon, Marko Gjorgjievski, Iustin Moga, Bill Ristevski

**Affiliations:** 1 Division of Orthopaedic Surgery McMaster University Hamilton, ON Canada; 2 Centre for Evidence-Based Orthopaedics McMaster University Hamilton, ON Canada; 3 Division of Orthopedic Surgery Dalhousie University Halifax, NS Canada

**Keywords:** distracted driving, driver distraction, driving while distracted, inattentive driving, Google, car distractions, texting and driving, cell phones

## Abstract

**Background:**

Distracted driving has become alarmingly widespread, and its prevalence continues to increase despite efforts by government and nongovernment organizations to educate the public about this pervasive problem. Every year, 1.35 million people die, and nearly 80 million people get injured in road traffic incidents. Motor vehicle crashes are the leading cause of death among young people, and distracted driving plays a huge role in road traffic fatalities and injuries. Considering that most people now use the internet as an information source and Google is the most visited website and number one online search engine in the world, we performed a qualitative analysis of information available through Google on distracted driving and its outcomes.

**Objective:**

The goal of this study was to analyze the quality and accuracy of the information on distracted driving and its consequences available to the general public when using Google as a search engine for distracted driving.

**Methods:**

In November 2018, a nonregional Google search on distracted driving was conducted. The first two pages of the Google search results were selected for analysis. Data were collected on the type of website, type of distraction, consequences of distracted driving described, presence and referencing of statistics, and orthopedic and nonorthopedic injuries described, with their acute and chronic sequelae.

**Results:**

In total, we analyzed 25 websites: 12 websites (48%) were from government bodies, which were the most common type of websites; 19 (76%) of the sites provided statistics; and 15 (60%) referenced the source of the statistic. Mobile phones were the most frequently cited type of distraction, with 17 (68%) sites discussing it, while death was the most commonly mentioned consequence of distracted driving, quoted in 15 (60%) of the websites. Additionally, 52% of the sites provided tips on how to avoid distracted driving. Only one website mentioned orthopedic injuries.

**Conclusions:**

The prevalence of distracted driving is increasing, and so are the consequences associated with it. Nevertheless, the information available online does not accurately describe the current circumstances regarding this issue. The National Highway Traffic Safety Administration attributed 391,000 injuries and 3477 deaths to distracted driving in 2015, which are 5000 more injuries and almost 150 more fatalities compared to 2011. However, despite these figures, most of the websites discussed death as a consequence of distracted driving and often overlooked injuries, even though injuries are over 100 times more likely to occur in distraction-affected crashes. The websites also largely fail to address other forms of driving distractions, like daydreaming or talking to a passenger, and mostly focus on mobile phone–related activities as distractions. More specific information on the dangers of distracted driving and nonlethal trauma may support an overall cultural shift to curb this behavior.

## Introduction

Distracted driving is not only dangerous but also very common and has become an epidemic in North America. In the United States, 37,461 people were killed in motor vehicle crashes (MVCs) in 2016. Of these, 3450 (9%) deaths were caused by distracted driving [1]. Additionally, in the previous year, nearly 2.5 million people were injured in MVCs, and approximately 16% of those injuries were a result of distractions [1]. The gravity and pervasiveness of the growing threat of distracted driving have prompted the World Health Organization to identify it as one of the priorities in global road safety. [2]

Public opinion research suggests that distracted driving was already considered a problem in 2011. A survey conducted in Iowa that year showed that 98% of respondents believed distracted driving to be a serious or somewhat serious threat to traffic safety [3]. However, despite this opinion, distracted driving remains prevalent [4]. A Canadian survey completed in 2018, found that 69% of the participants think the most distracting activity while driving is using a mobile phone, and yet, according to the same study, 51% of the respondents still communicate on their mobile phones at least once a week while driving [5]. Data from the National Collision Database, which show a continuous increase in injuries and deaths due to distracted driving, further support the fact that drivers continue to engage in this unsafe practice despite the evident risks [6].

Activities such as talking with a passenger, daydreaming, eating or drinking, programming the navigation systems, and using mobile phones while driving, which cause the driver to divert their attention away from safely operating the vehicle, are all forms of distracted driving [7]. Mobile phones, in particular, have been found to have a greatly negative effect on driving performance [8], which places the driver at significant risk for a MVC. In fact, the odds of crashing when making a call on a handheld device increases by 12 times [9], and the crash risk for texting while driving is almost double that (23 times) [10].

Distracted driving behavior may be due to a lack of appropriate education on distracted driving. Many people may inherently comprehend that distracted driving is a threat to public safety, but they may lack specific information that could reinforce safer behavior. The majority of people today turn to the internet as their primary research/education tool when wanting to gain new knowledge on a topic such as distracted driving. Google is both the most commonly used data search engine and the most visited website in the United States and globally [11]. Of all searches performed on a desktop or laptop, 76% are through Google, and the figures are even higher for mobile phones, where more than 85% of the searches go through this engine [12]. Google processes more than 63,000 search requests per second, which equates to more than 5.4 billion queries a day [13]. Therefore, it is reasonable to assume that the preferred online research method for the majority of people will be through this platform.

Although multiple websites provide information on distracted driving to the general public, little is known about what information these websites offer regarding the specific causes and outcomes of distracted driving. Using modern internet-based methods in research to assess the available information on distracted driving could bring us closer to understanding why this risky behavior is so prevalent. The primary goal of this study is to qualitatively analyze the information available to the general public on websites for distracted driving and assess the quality and accuracy of that information. The secondary goal is to specifically examine whether information about potential injuries is conveyed.

## Methods

Using a nonregional Google search setting, a search on the phrase “distracted driving” was conducted.The search was performed in November 2018, and the first two pages of the Google search results were selected for analysis. We collected data on the type of the website, consequences of distracted driving, type of distraction, presence of statistics, and referencing of statistics as well as orthopedic and nonorthopedic injuries together with their acute and chronic manifestations. We categorized the websites as government, nonprofit, private company, and sites belonging to a foundation. Distracted driving consequences were described as death/fatality, injury, and legal repercussions. Driving distractions presented in the websites were assigned as distractions involving mobile phones, hands-free mobile phones, infotainment, and others (passengers, food/drink, reaching, and outer-vehicle distractions).

## Results

In total, we analyzed 25 websites. The most common search results were government body websites, which accounted for 12 websites (48%). Additionally, seven (28%) websites were run by nonprofit organizations, followed by five (20%) that were run by private company websites, and one (4%) that was a foundation-owned webpage (Table 1). Government websites included multiple state departments of transportation as well as US federal agencies such as the National Highway Transportation Safety Administration (NHTSA) and the Federal Communications Commission. Nonprofit organizations included the American Automobile Association, National Safety Council, the Governors’ Highway Safety Association, and a pediatric hospital-associated program that helps parents monitor their children’s whereabouts using mobile device tracking (TeenSafe). One website (Enddd.org) is the project of a memorial foundation established in the name of a distracted driving victim.

**Table 1:** Types of websites for distracted driving.

Types of websites	Value, n (%)
Government	12 (48)
Nonprofit	7 (28)
Private company	5 (20)
Foundation	1 (4)

Most websites described multiple types of distractions (Table 2). The most common distraction cited in 17 sites was mobile phone use (68%), followed by vehicle entertainment and information systems discussed in eight of the sites (32%). Seven (28%) of the reviewed webpages mentioned food, drink, other passengers, and outer-vehicle distractors, and three of the websites (12%) distinguished hands-free telecommunications as a significant distraction. Finally, seven websites (28%) did not identify any specific distractions.

**Table 2:** Distractions described in websites for distracted driving. Many websites identified multiple distraction types.

Distractions	Websites describing distraction, n (%)
Mobile phones (includes texting)	17 (68)
Mobile phones (distinguishes hands-free)	3 (12)
Infotainment system	8 (32)
Other distractions (passengers, food, drink, reaching for object, outer vehicle)	7 (28)
Broad statement/nonspecified	7 (28)

Of the websites analyzed, 19 (76%) included distracted driving statistics and 15 (60%) also provided references for those statistics. The most commonly referenced data were those published by the NHTSA. The Traffic Safety Facts report published by NHTSA found that 3450 deaths and over 391,000 injuries were attributable to distracted driving in 2016.

In terms of the consequences of distracted driving, most websites listed multiple outcomes (Table 3). The most frequently identified consequence was death, cited in 15 (60%) of the sites. Although “injury” (or “injuries”) was listed in 11 (44%) websites, only one (4%) of these alluded to specific bodily systems affected by the injury. Legal repercussions due to distracted driving including fine, demerit points, indictment, and incarceration were listed in 6 (24%) websites.

**Table 3:** Consequences described in websites for distracted driving. Many websites identified multiple consequences.

Consequences	Websites Describing Consequence n (%)
Death/mortality/fatality	15 (60)
Legal repercussions	6 (24)
Injury	11 (44)
Injury specified (orthopedic, nonorthopedic)	1 (4)

## Discussion

### Principal Results

This study qualitatively analyzed 25 websites on distracted driving using Google as a search engine. The findings of this analysis demonstrate notable differences between the way distracted driving is portrayed on the internet versus reality. For example, the results of this study show that death was the most common consequence of distracted driving presented on the websites. In addition, 15 (60%) websites cited a fatality as an outcome, compared to injuries that were reported on 11 (44%) of the sites. Depending on the source, current data available on road traffic accidents resulting from distracted driving proves that injuries outnumber death as a consequence, with a ratio of 60-113 to 1 [14,15].

The study also demonstrated discrepancies between the online and actual representations of various driving distractions. Mobile phones and texting, discussed in 17 (68%) of the sites, were shown to be the most common distracting activities. However, a report by Erie Insurance completed using data from the Fatality Analysis Reporting System lists daydreaming as the number one distraction involved in 61% of fatal MVCs and ranked mobile phones second at 14% [16]. Another report from the NHTSA indicates that talking with a passenger is the most common distracting activity, responsible for 57% of distracted driving collisions, while combined phone use accounted for 11% [17].

The fact that texting is the most common type of distraction described is not surprising. Gallup [18] reported that texting is now the preferred form of communication among Americans under the age of 50 years and that the prevalence of texting is higher among younger people in the United States. The Centers for Disease Control and Prevention found that drivers aged 16-19 years are at highest risk of being involved in MVCs [19], and the NHTSA reported that the same age group also had the highest percentage of distracted drivers involved in fatal MVCs [1]. This could be the basis of why websites for distracted driving have focused on mobile phone use so heavily.

As expected, government agencies publish the majority of information available to the public concerning distracted driving. This finding is encouraging, as it suggests that government authorities are actively working to address this problem.

Although many websites provided statistics and consequences and 11 websites mentioned generic injuries, it is worrisome that only one website cited orthopedic injuries resulting from distracted driving. The Canadian National Trauma Registry reports that 79% of the major injuries in hospitalized patients are musculoskeletal in nature and that car crashes are the number one cause of major injuries [20]. Considering that at least 16% of all injuries sustained in an MVC are distraction related [14], it is logical to assume that orthopedic injuries would make up the majority of these injuries. Unfortunately, this outcome of distracted driving is massively ignored by these websites.

Many of the reviewed websites offered guidance on how to prevent distracted driving. However, all of these invoked driver self-discipline and other active mechanisms to enforce behavior modification (eg, “Just Drive”). Likely, decreasing distracted driving will be a combination of education, cultural shift, and passive restraints, for example, mobile phone apps restricting phone use while driving [21], collision avoidance, and self-driving technology in vehicles.

### Limitations

The main limitation of this study is that little is publicized about the algorithm for the google.com/ncr search; thus, it is unknown exactly how website hits are generated on Google. However, by using a nonregional Google search, we attempted to collect as many websites as possible without the bias of regional preferences. Therefore, it is likely that our search results represent what the average information seeker would find without any filters set. Additionally, the primary strength of this study is that it examines a contemporary public safety issue using the most popular search engine.

### Comparison With Prior Work

According to the World Health Organization, almost 1.35 million people die in MVCs every year, and for every one of those traffic fatalities, 60 people get injured [15,22]. Distracted driving is a considerable contributor to these figures and continues to be a common practice among the drivers despite attempts by many organizations to control this pervasive behavior through education. It seems to be especially widespread among the younger drivers [23-25], as it has become the leading cause of death for teenagers [26].

Many North American jurisdictions passed antidistracted driving laws to curb this problem. Unfortunately, despite introducing new legislation, distracted driving remains rampant today. The Alberta Government in Canada released data that show no decrease in distracted driving convictions in the 4 years since the distracted driving legislation was passed (n=25,958 in 2012 and n=27,281 in 2016) [27]. Moreover, in Ontario, apart from 2012, distracted driving has been the leading cause of MVC fatalities since 2009, when legislation prohibiting the use of handheld devices was first introduced [28]. The NHTSA also continues to report an alarming number of collisions due to distracted driving, with no signs of this problem ending: 885,000 crashes in 2015 compared with 826,000 in 2011 [14].

Google is the most visited website globally [11], and with 92.37% of the market share and more than 5.6 billion queries a day, it is the most popular search engine in the world [13,29]. It is available in 149 different languages [30] and operates in more than 200 countries, making it a particularly useful tool when researching topics of interest online. Studies have examined Google’s increasing involvement in the search for medical information online and have found that despite some deficiencies, it can be an effective medium [31]. Research looking into the information on various medical issues available on the internet using Google as a search engine includes subjects such as views on vaccination [32], psychoactive agents [33], fractures [34], and skin cancer [35].

Although distracted driving has become increasingly common, and interest on this subject is high, there is limited research on the information available online regarding this issue. To our knowledge, so far, there have not been any publications concerning information on the internet on distracted driving, which are accessible through Google. A recent study [36] completed at the authors’ home institution, which is currently in preprint, has examined the messages represented in distracted driving videos on YouTube with similar findings.

### Conclusions

Distracted driving is becoming more prevalent, and with it, so are the injuries and loss of lives associated with this problem. Our Google search project found that little to no specific information is available to the general public regarding types of injuries, including the potential disability resulting from them. Conversely, death due to distracted driving, which, in reality, occurs much less often than injuries, is the most commonly presented outcome in these websites. Unfortunately, websites focusing mainly on fatality secondarily to distracted driving eclipses the reality of millions of people surviving distraction-related MVCs who continue to live with chronic pain, disabilities, decreased quality of life, and increased financial burden. Furthermore, the sites mostly focus on mobile phones as potential distractions and largely neglect other more common forms of distracted driving. Although the general public recognizes that distracted driving is dangerous, in principle, the lack of specific information on the consequences of this behavior may be contributing to its continued practice.

## Abbreviations

MVC: motor vehicle crash
NHTSA: National Highway Traffic Safety Administration
